# Tranexamic acid in multiply injured patients–the independent risk of thromboembolic complications with repeated dosing: retrospective analysis based on the TraumaRegister DGU®

**DOI:** 10.1186/s13049-025-01512-1

**Published:** 2025-11-15

**Authors:** Jörg Bayer, Tim Kirchner, Rolf Lefering, Lisa Bode, Hagen Schmal, Ferdinand C. Wagner

**Affiliations:** 1 Department of Orthopaedic and Trauma Surgery, Medical Center, Faculty of Medicine, University of Freiburg, Hugstetter Straße 55, Freiburg, 79106 Germany; 2 Department of Orthopaedic and Trauma Surgery, Schwarzwald-Baar Hospital, Klinikstraße 11, Villingen-Schwenningen, 78052 Germany; 3 Faculty of Health, IFOM-Institute for Research in Operative Medicine, University Witten/Herdecke, Ostmerheimer Straße 200, Cologne, 51109 Germany; 4 Department of Orthopedic Surgery, University Hospital Odense, Sdr. Boulevard 29, Odense, 5000 Denmark

## Abstract

**Background:**

Tranexamic acid is an established drug in the treatment of bleeding trauma patients. Concerns have been raised over possible complications of tranexamic acid regarding thromboembolic events as serious complications during the treatment of severely injured patients.

**Methods:**

In our study we retrospectively analyzed data from 2015—2019 of multiply injured patients receiving tranexamic acid during distinguished treatment periods from the TraumaRegister DGU®. We statistically analyzed overall thromboembolic complications during hospital stay in the context of number of single-dose tranexamic acid administrations.

**Results:**

We report on 37,342 patients, of whom 1,151 (3.1%) suffered from thromboembolic events. Patients without tranexamic acid treatment suffered from thromboembolic events in 2.3%, prehospital and emergency department administration increased the incidence to 4.8% and 5.2%, respectively. Administering tranexamic acid twice or three times was associated with an increased incidence of 8.5% and 8.2%, respectively. In a multivariate logistic regression, we uniquely show an independently associated risk for thromboembolic complications with every consecutive administration of tranexamic acid (one application: odds ratio (OR) 1.56, *p* < 0.001; two applications: OR 1.79, *p* < 0.001; three applications: OR 1.50, *p* = 0.113).

**Conclusions:**

In our study we report on an associated risk of thromboembolic events in multiply injured patients with every single time tranexamic acid was administered in our study. Thus, before a repetitive dose of tranexamic acid is administered checking for indication is advised and especially in multiply injured patients receiving repeated administrations of TXA starting a thromboprophylaxis, as soon as possible after the traumatic bleeding disorder is controlled, is important.

## Background

Venous thromboembolism (VTE) is a relevant complication during the treatment of severely injured patients and the incidence of VTE is reported to be as high as 8% [1]. Several risk factors (e.g. older age, obesity, male sex, higher Injury Severity Score (ISS), pelvic injury, lower extremity injury, spinal injury, delayed VTE prophylaxis) have been established [1, 2] and its occurrence is associated with increased morbidity and mortality [3]. While VTE pose a concomitant risk to multiply injured patients during the entire treatment period [1], traumatic hemorrhage is the most common cause of early death in the injured and approximately 25% of trauma victims have immediate coagulative malfunction and up to 40% die from hemorrhagic shock [4, 5]. Acute traumatic coagulopathy aggravates hemorrhage, increases mortality and is commonly associated with hyperfibrinolysis [6, 7]. Tranexamic acid (TXA) is an antifiber solvent that can improve clot stability by inhibiting plasminogen activation and fibrinolysis; thus, it may be an effective treatment [8].

The effect of in-hospital administration of TXA in patients with trauma was evaluated in the Clinical Randomisation of an Antifibrinolytic in Significant Haemorrhage (CRASH)–2 trial where TXA has proven to reduce 28-day mortality among patients with suspected bleeding [9]. Subsequent studies confirmed the aforementioned results [10, 11] and the administration of TXA is recommended in current international guidelines [12, 13].

Furthermore, it has been shown that administration of TXA in severe bleeding is most effective, when given immediately after bleeding onset [14]. In trauma associated hemorrhage administration of TXA has not only shown to reduce 28-day mortality among patients admitted to a hospital with suspected bleeding (CRASH-2 trial) [9], but the administration of TXA in hemorrhagic patients within 3 h of injury significantly reduced the risk of death due to bleeding [15]. Since earlier treatment proved to be more effective [15] studies evaluated the treatment of polytraumatized patients in the prehospital setting [16–20]. Although the reduction of early mortality due to hemorrhage after hospital admission was often reported in TXA treated severely injured patients [16–20] these results were not unequivocally presented by others [21–23]. Additionally, concerns have been raised as to the safety of TXA, while some reports consider TXA safe [24–26], reports of significant incidences of VTE in severely injured patients have been published and independently attribute these VTE events to TXA [1, 20, 22, 27]. An explanation might be that 3 different phenotypes of fibrinolytic responses of the body to massive trauma have been described: hyperfibrinolysis, physiologic fibrinolysis, and hypofibrinolysis (also referred to as “fibrinolytic shutdown” [28]. Thus, the non-discriminative and liberal administration of an antifibrinolytic agent to a patient population that could be largely in a hypofibrinolytic state does not seem to be physiologically or pharmacologically sound and would potentially expose patients to a higher risk for thromboembolic complications [29]. Apart from the discussion whether distinct patient populations might not benefit from or will be even potentially harmed by TXA administration, there have been reports on possible dose dependent risks for VTE in trauma patients receiving TXA [30, 31]. These reports consider different quantities of a single dose or intervals between doses of TXA. Nevertheless, there are situations where hemorrhagic trauma patients might be repeatedly treated with TXA, e.g. prehospital, trauma room, operation theater and/or intensive care unit.

This is why we proposed our study to investigate the potential risk for VTE with repeated applications of TXA in a large cohort of multiply injured patients. We hypothesized that, in multiply injured patients, the administration of TXA constitutes an independent risk factor for VTE and the risk for VTE is also depending on the timepoint (prehospital, trauma room, intensive care unit) and the number of consecutive TXA administrations.

## Methods

### TraumaRegister DGU®

The TraumaRegister DGU® (TR-DGU) of the German Trauma Society (Deutsche Gesellschaft für Unfallchirurgie, DGU) was founded in 1993 and represents a multi-center database containing pseudonymized and standardized documentation of severely injured patients. Data are collected prospectively in 4 consecutive phases: A) pre-hospital phase, B) emergency room and initial surgery, C) intensive care unit and D) discharge. The documentation includes detailed information on demographics, injury patterns, comorbidities, pre-hospital and clinical management, intensive care course, key laboratory findings including transfusion data, and outcome. The inclusion criterion is admission to hospital via the emergency room with subsequent monitoring in intensive or intermediate care, or arrival at hospital with vital signs and death before admission to intensive care unit. The infrastructure for documentation, data management and data analysis are provided by the AUC—Academy for Trauma Surgery (AUC—Akademie der Unfallchirurgie GmbH), which is affiliated to the German Trauma Society. The scientific leadership is provided by the Committee on Emergency Medicine, Intensive and Trauma Management (Sektion NIS) of the German Trauma Society. Participating clinics enter their pseudonymized data into a central database via a web-based application. Scientific analyses are approved according to a peer review process defined in the publication guidelines of the TR-DGU. The participating clinics are mainly located in Germany (90%), but an increasing number of clinics from other countries are also contributing data (currently from Austria, Belgium, China, Finland, Luxembourg, Slovenia, Switzerland, the Netherlands and the United Arab Emirates). Currently, about 38,000 cases from nearly 700 clinics are entered into the database each year. Participation in the TR-DGU is on a voluntary basis. It is mandatory for TraumaNetzwerk DGU® clinics to enter at least one basic data set for quality assurance purposes.

This retrospective multicenter cross-sectional study evaluated the data of all severely injured in the TR-DGU from 01.01.2015 to 31.12.2019. The study strictly adheres to the publication guidelines of the TR-DGU and is registered under the TR-DGU project ID 2020–036. The research project has been approved by the local ethics committee (University of Freiburg, 22–1007-retro).

### Definition and documentation of thromboembolic events

Clinically relevant VTE events included deep vein thrombosis (DVT), pulmonary embolism (PE), myocardial infarction (MI) and stroke. Thrombosis of a superficial vein or upper extremity is defined as “other”. VTE events after hospital discharge are not part of the documentation of the TR-DGU and therefore are not included in this study.

### Definition and documentation of timepoint, dosage of TXA and injury severity

To examine the influence of singular or multiple TXA administration in multiply injured patients on VTE complications we distinguished between

1. administration in the prehospital setting
2. in the emergency department (ED; defined as the time interval from hospital arrival and early operative phase until admission to the intensive care unit (ICU))
3. during the first 48 hours on the intensive care unit ICU

As TXA administration is either documented as “yes” or “no” in the TR-DGU we assumed an administration in line with the recommendations of national and international guidelines.

Overall injury severity was calculated according the Injury Severity Score (ISS) [32].

### Population, inclusion and exclusion criteria

TraumaRegister DGU® datasets of multiply injured patients treated between 2015 and 2019 in Austria, Germany or Switzerland were analyzed. Patients were included in our analysis if records were complete with regard to the documentation of VTE events and TXA administration. Consequently, patients documented with basic data set only (no VTE events) were not included in this study.

**Fig. 1:**
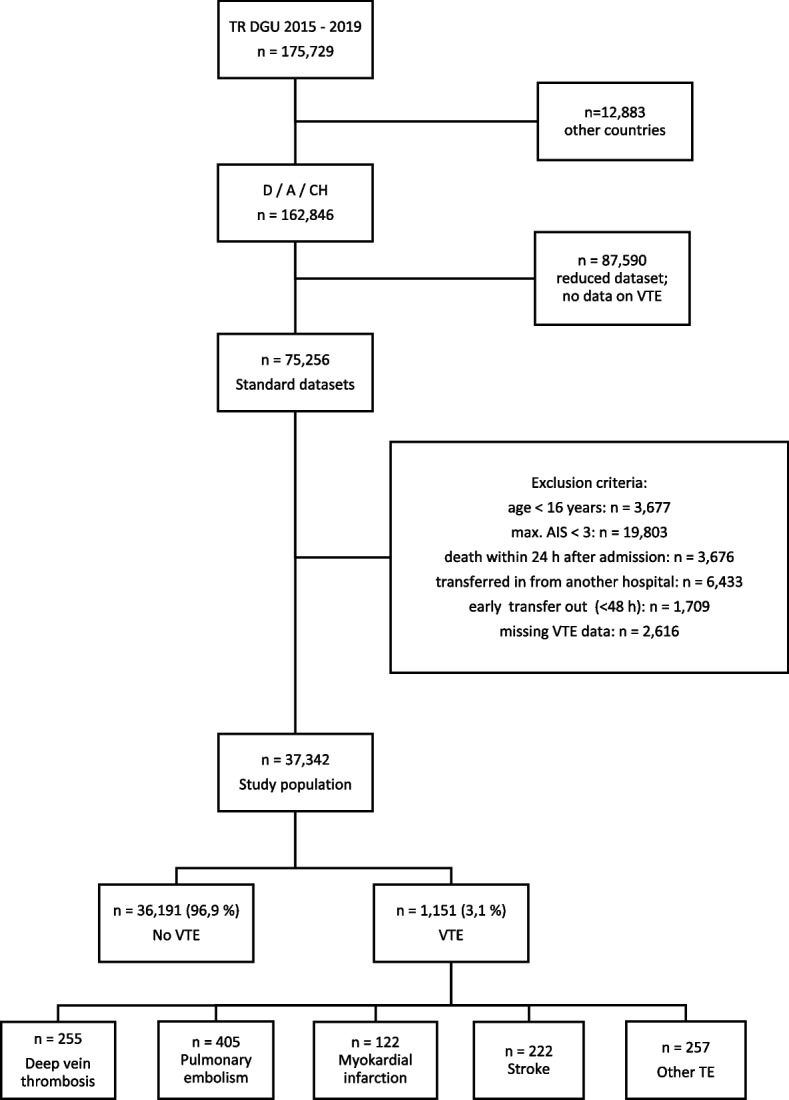
Flowchart of study patients. Patients treated in Austria (A), Germany (D) or Switzerland (CH) were analyzed. Some patients suffered from multiple VTE

The following exclusion criteria were applied: age < 16 years, maximum Abbreviated Injury Score (AIS) < 3, early death (defined as death occurring within the first 24 h after hospital admission), patients transferred in (defined as patients treated in a different hospital prior to admission) or early transfers out (defined as patients being transferred to another hospital within 48 h after initial admission). (Fig. 1).

### Data analysis

Continuous variables are shown as mean ± standard deviation (SD) or median and interquartile range (IQR), depending on the distribution, and incidence rates as percentages, respectively. Differences in the study population were compared with the χ^2^-test for categorial variables and the Mann–Whitney U-test for continuous variables, respectively.

To identify independent risk factors for the development of VTE complications a multivariable logistic regression analysis was performed. The analyzed predictors are summarized in Table 1*.* Odds Ratios (OR) are presented with 95% confidence intervals (CI_95_). Nagelkerke's R^2^ was used to describe the predictive power of the model.

**Table 1:** Demographics of patients with (VTE) and without VTE (Non-VTE)

	Non-VTE *n* = 36,191	VTE *n* = 1,151	*p*—value
Age (years; mean, SD)	53 (21)	57 (20)	< 0.001
Age ≥ 60 years	14,379 (39.7%)	541 (47.0%)	< 0.001
Male gender	25,843 (71.4%)	864 (75.1%)	0.007
Timepoint of TXA			
- None	27,884 (77.0%)	671 (58.3%)	< 0.001
- Prehospital	2,808 (7.8%)	142 (12.3%)	
- ED	4,503 (12.4%)	246 (21.4%)	
- prehospital & ED	773 (2.1%)	72 (6.3%)	
- Prehospital, ED & ICU	223 (0.6%)	20 (1.7%)	
Doses of TXA			
- 1	7,235 (20.0%)	370 (32.1%)	< 0.001
- 2	1,803 (5.0%)	144 (12.5%)	
- ≥ 3	223 (0.6%)	20 (1.7%)	
Blood transfusion			
- none	32,820 (90.7%)	871 (75.7%)	< 0.001
- 1–9 PRBC	3,007 (8.3%)	223 (19.4%)	
- ≥ 10 PRBC	364 (1.0%)	57 (5.0%)	
PPSB administration	1,807 (5.0%)	145 (12.6%)	< 0.001
Fibrinogen administration	3,276 (9.1%)	259 (22.5%)	< 0.001
ISS ≥ 16	24,189 (66.8%)	978 (85.0%)	< 0.001
Pre-injury ASA 3/4	5,770 (15.9%)	246 (21.4%)	< 0.001
Coagulopathy (Quick ≤ 60 or INR ≥ 1.4 or PTT ≥ 40)	4,035 (11.5%)	211 (19.1%)	< 0.001
Pre-injury anticoagulation	5,494 (15.2%)	225 (19.5%)	< 0.001
Hb < 8 mg/dl	893 (2.5%)	65 (5.7%)	< 0.001
Blunt injury mechanism	33,438 (95.8%)	1,062 (95.9%)	0.850
Accident mechanism			
- Car	6,993 (19.5%)	232 (20.3%)	0.384
- Motorbike	4,978 (13.9%)	162 (14.2%)	
- Bicycle	3,606 (10.0%)	98 (8.6%)	
- Pedestrian	2,123 (5.9%)	71 (6.2%)	
- High fall (> 3 m)	5,691 (15.9%)	184 (16.1%)	
- Low fall (< 3 m)	8,555 (23.8%)	253 (22.2%)	
ISS (mean, SD)	20.9 (10.5)	26.9 (12.7)	< 0.001
Serious injuries (AIS ≥ 3)			
- Head	15,562 (43.0%)	608 (52.8%)	< 0.001
- Thorax	17,560 (48.5%)	592 (51.4%)	0.052
- Abdomen	4,295 (11.9%)	226 (19.6%)	< 0.001
- Extremities	10,940 (30.2%)	49 (39.0%)	< 0.001
Shock (SBP ≤ 90 mmHg)			
- prehospital	2,558 (8.1%)	150 (84.8%)	< 0.001
- ED	2,661 (7.7%)	175 (16.0%)	< 0.001
Catecholamines (yes/no)	6,816 (18.8%)	446 (38.7%)	< 0.001
Organ failure	10,762 (32.3%)	754 (68.4%)	< 0.001
Multiple organ failure	6,214 (18.6%)	550 (49.9%)	< 0.001
Lung failure	4,108 (12.3%)	376 (34.1%)	< 0.001
Sepsis	2,065 (6.2%)	250 (23.0%)	< 0.001
Number of operative procedures (median, IQR)	1 (0–3)	3 (1–7)	< 0.001
Prehospital infusion > 1000 mL	4,522 (12.5%)	247 (21.5%)	< 0.001
Infused volume prehospital (mL; mean SD)	736 (563)	908 (710)	< 0.001
Infused volume in the ED (mL; mean SD)	1,237 (1661)	2,144 (2569)	< 0.001
Mechanical ventilation (days; median, IQR)	0 (0–3)	6 (1–18)	< 0.001
LOS on ICU (days; median, IQR)	3 (1–10)	13 (5–26)	< 0.001
LOS in hospital (days; median, IQR)	14 (8–23)	26 (15–42)	< 0.001
In-hospital mortality	2,704 (7.5%)	252 (21.9%)	< 0.001

*AIS* Abbreviated Injury Scale, *ASA* Classification of American Society of Anesthesiologists, *ED* emergency department, *ICU* intensive care unit, *IQR* interquartile ratio, *ISS* Injury Severity Score, *LOS* length of stay, *OF* organ failure, *PRBC* packed red blood cells, *SBP* systolic blood pressure, *SD* standard deviation, *TXA* tranexamic acid

The level of significance was set at *p* < 0.05. All data were analyzed using SPSS, version 29.0 (IBM Corp. New York, USA).

## Results

### Population

The TR-DGU database of patients treated between 2015 and 2019 consists of 175,729 datasets. After applying the aforementioned inclusion and exclusion criteria 37,342 datasets were available for final analysis. (Fig. 1).

Briefly, our study population consists of trauma patients aged ≥ 16, suffering from at least one serious injury (AIS ≥ 3) and surviving the first 24 h after hospital admission.

### Patient characteristics

Demographic characteristics of patients with thromboembolic (VTE) and without thromboembolic (Non-VTE) complications are depicted in Table 1.

Our population is homogenous concerning mechanism of injury/accident. Significant differences are observed regarding age, gender, pre-existing medical conditions, coagulopathy (Quick’ value ≤ 60% or INR ≥ 1.4 or PTT ≥ 40 s), pre-injury anticoagulation, anemia (Hb < 8 mg/dl), overall injury severity (ISS), head and abdominal injury as well as injury to the extremities, incidence of hemorrhagic shock and need for massive transfusion and need for catecholamines. No significant differences were observed among patients with and without VTE complications regarding a serious injury to the thorax (AIS_Thorax_ ≥ 3).

Detailed data and *p*-values for each parameter are documented in Table 1.

Patients suffering from thromboembolic events had higher morbidity and mortality rates. In-hospital mortality was 21.9% for the VTE-group compared to 7.5% in the group without thromboembolic events (*p* < 0.001). Duration of mechanical ventilation, days spent in ICU as well as overall hospital length of stay were significantly longer among patients suffering from VTE compared to patients without a thromboembolic complication. Rates of organ failure and sepsis were also significantly higher in patients suffering from VTE compared to Non-VTE.

Detailed data and *p*-values for each parameter are documented in Table 1.

### Thromboembolic events

One thousand, one hundred fifty-one of 37,342 patients suffered from a thromboembolic event (3.1%). Pulmonary embolism (PE) accounted for 405, deep vein thrombosis (DVT) for 255, myocardial infarction (MI) for 122 and stroke for 222 cases, respectively. Other TE occurred in 257 patients. The summary of all individual VTE cases is higher than 1,151 since some patients suffered from multiple VTE. All VTE incidences are shown in Fig. 1.

### Influence of number of TXA administrations on thromboembolic events

**Fig. 2:**
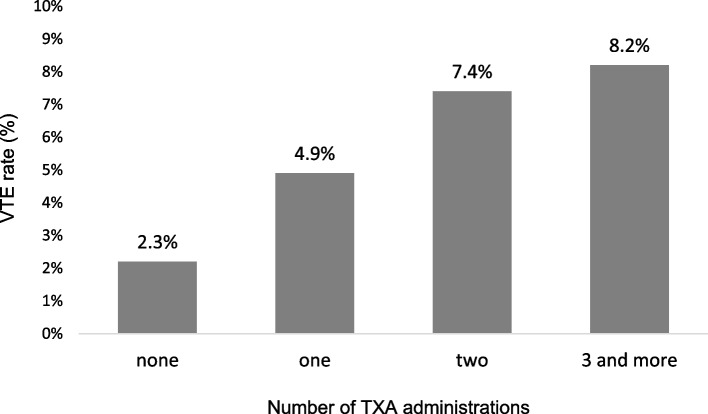
Relationship between number of TXA administrations and thromboembolic incidences

A total of 8,787 patients (23.5%) received at least one dose of TXA. With every dose of TXA the incidence of VTE increased in our population. As depicted in Fig. 2, multiply injured patients without TXA suffered from VTE in 2.3% of cases, whereas one dose of TXA was associated with an increase of the incidence of thromboembolic complications to 4.9%, two doses to 7.4% and ≥ 3 administrations to 8.2%, respectively.

### Influence of timepoint of TXA administration on the incidence of thromboembolic events

The influence of the moment of TXA administration in multiply injured patients on VTE complications was examined by distinguishing between administration in the prehospital setting, the emergency department (ED) and the intensive care unit (ICU). Most severe trauma patients received no TXA at all (76.4%). Among TXA administrations, most patients received TXA once in the ED (12.7%), but a single prehospital administration was also frequent (7.9%). We were able to identify 1,088 patients who received more than one dose of TXA, mostly twice (prehospital + ED), but three consecutive administrations were also recorded sometimes (0.7%).

Table 2 demonstrates the relationship between time of administration and the incidence of VTE events in multiply injured patients. VTE incidence in patients without TXA administration was 2.3%. Irrespective of the number of TXA administrations, the VTE incidence in the TXA-treated group overall was 5.5%. Prehospital only administration of TXA was associated with a thromboembolic incidence in 4.8%, ED only in 5.2%, respectively. The administration of TXA at both times, prehospital and in the ED, resulted in a related increased incidence of 8.5% while an administration in the prehospital setting, the ED and additionally the ICU resulted in a VTE incidence of 8.2%.

**Table 2:** Incidence of VTE and time of TXA administration

	No TXA	Prehospital only	ED only	Prehospital and ED	Prehospital, ED and ICU
Cases	28,555	2,950	4,749	845	243
	76.4%	7.9%	12.7%	2.3%	0.7%
VTE	671	142	246	72	20
	2.3%	4.8%	5.2%	8.5%	8.2%

Number of patients (cases) treated without, with single or multiple TXA administrations during different treatment periods and number of patients suffering from VTE

*ED* emergency department, *ICU* intensive care unit, *TXA* tranexamic acid

### Multivariate logistic regression model

Univariate analysis identified certain risk factors increasing the risk for the developing thromboembolic events (Table 1). To determine whether the above identified factors serve as independent risk factors for VTE a multivariate logistic regression model with VTE complication as the dependent variable was conducted among the 37,342 patients. The results are shown in Table 3.

**Table 3:** Multivariate logistic regression analysis with VTE as dependent variable

Predictor	n	OR	95% CI for OR	p
Age (reference: < 60)				
60—69	5,339	1.49	1.26–1.77	< 0.001
70—79	5,021	1.57	1.31–1.87	< 0.001
80 and older	4,560	1.34	1.09–1.65	0.010
Male gender	26,707	1.35	1.13–1.50	< 0.001
Pre-existing diseases (ASA 3/4)	6,016	1.35	1.14–1.60	< 0.001
Injury Severity Score (per point)	37,342	1.02	1.01–1.03	< 0.001
Head injury (AIS 3 +)	16,170	1.19	1.01–1.40	0.041
Thoracic injury (AIS 3 +)	18,152	0.88	0.76–1.01	0.073
Abdominal injury (AIS 3 +)	4,521	1.26	1.06–1.51	0.010
Extremity injury (AIS 3 +)	11,389	1.22	1.05–1.41	0.008
Unconsciousness (GCS 3–8)	6,191	1.19	1.02–1.39	0.025
Shock (SBP ≤ 90 mmHg)	4,636	1.09	0.89–1.32	0.410
Prehospital volume > 1000 ml	4,769	1.23	1.05–1.44	0.012
Blood transfusion (reference: none)				
1–9 units of PRBC	3,230	1.37	1.12–1.68	0.002
mass transfusion ≥ 10 units)	421	2.22	1.55–3.19	< 0.001
Coagulopathy	4,246	0.90	0.75–1.07	0.239
Administration of PPSB	1,952	1.02	0.81–1.28	0.852
Administration of Fibrinogen	3,535	1.15	0.93–1.42	0.187
Tranexamic acid (reference: none)				
1 administration	7,605	1.56	1.35–1.81	< 0.001
2 administrations	1,947	1.79	1.43–2.24	< 0.001
3 administrations	243	1.50	0.91–2.48	0.113

Total patients *n* = 37,342

Shock state includes prehospital and/or ED admission SBP ≤ 90 mmHg recorded

*AIS* Abbreviated Injury Scale, *ASA* Classification of American Society of Anesthesiologists, *CI* confidence interval, *GCS* Glasgow Coma Scale, *OR* odds ratio, *PRBC* packed red blood cells, *SBP* systolic blood pressure

Strong independent risk factors for the development of VTE were blood transfusion (especially mass transfusion ≥ 10 PRBCs, OR 2.22, *p* < 0.001), TXA administration (one application: OR 1.56, *p* < 0.001; two applications: OR 1.79, *p* < 0.001; three applications: OR 1.50, *p* = 0.113), age ≥ 60 years (60—69 years: OR 1.49, *p* < 0.001; 70—79 years: OR 1.57, *p* < 0.001; ≥ 80 years: OR 1.34, *p* = 0.01) and pre-existing diseases (ASA 3/4, OR 1.35, *p* < 0.001).

After blood transfusion, the administration of TXA had the second most important effect on VTE development.

A weaker, but likewise positive relationship is documented for male gender, higher ISS (OR 1.02 per point of ISS increase), head injury (AIS ≥ 3), abdominal injury (AIS ≥ 3), extremity injury (AIS ≥ 3), unconsciousness (GCS 3–8) and need for prehospital volume administration > 1000 mL (all *p* < 0.05).

No correlation was found for thoracic injury (AIS ≥ 3, OR 0.88, *p* = 0.073) and shock (SBP ≤ 90 mmHg, OR 1.09, CI 0.89–1.32, *p* = 0.41).

Consistent with the above mentioned relatively small numbers of OR we report a Nagelkerke’s r^2^ of 0,061.

## Discussion

In this study we sought to investigate the potential risk for VTE with repeated applications of TXA in multiply injured patients.

Resuscitation after severe traumatic injury has undergone significant development over the past decade, with the use of early adjunctive strategies to prevent coagulopathy and improve hemostasis [33, 34]. One of these adjunctive measures is the application of TXA in hemorrhagic trauma patients [12, 13, 33]. Tranexamic acid has been hypothesized to mitigate hyperfibrinolysis and coagulopathy induced by shock and traumatic hemorrhage [29, 35]. A recent review on the effects of TXA on the immune system revealed that lysine receptor antagonist not only reduce fibrinolysis in trauma patients with severe hemorrhage but also exhibits pro-inflammatory or anti-inflammatory effects. These effects depend on factors such as the route of administration, dosage, duration since trauma onset, and the persistence of TXA treatment [36].

There has been an increasing number of hospital [9–11, 21, 22, 31, 37–40] and prehospital [16, 17, 19, 41–44] studies, as well as studies combining treatment intervals of TXA [45, 46], evaluating the use of tranexamic acid following severe traumatic injury.

In the mentioned studies above, evaluating the use of tranexamic acid following severe traumatic injury, TXA applications were reported in 99—10,096 patients, respectively. The majority of studies comprising < 500 patients. Only two studies report on > 1000 patients receiving TXA-treatment [9, 17]. In our collective 23.6% of our patients received at least one dose of TXA. Thus, we report on a large sample size, comprising 8,787 multiply injured patients who received at least one dose of TXA. On the other hand, in our study population 76.4% of patients received no TXA. Since almost 10% of our patients received packed red blood cells PRBC and 7% were in shock the indication for administering TXA in our retrospective population seems to had been reasonably weighed up.

Although many studies considered the application of TXA in trauma patients safe [24–26], a growing number of studies raised the issue of potential harm with higher incidences of VTE correlating with TXA application [1, 22, 31, 44, 47].

Available data regarding VTE incidences among trauma patients are inconsistent. These numbers strongly depend on the respective trauma patient collective, including injury mechanisms as well as injury severity. In a retrospective study from the National Trauma Data Bank (NTDB) of the American College of Surgeons incidences for DVT and PE were 1.06% and 0.42%, respectively [48]. Lichte et al. [49] published overall TE rates of 2.8% among 40,846 polytrauma patients. In a huge meta-analysis combining 30 studies, yielding almost 2 million patients, by Tran et al. the median VTE incidence in studies not using screening ultrasound was 3% [1]. We report on a 3.1% VTE incidence in our collective, which is consistent with the literature above.

The increased VTE risk among trauma patients is likely multifactorial related to venous stasis from immobilization, hypercoagulability from prothrombotic changes following tissue injury, and impaired fibrinolysis [50]. These findings support those well-established concepts and further elucidate the contribution of patient predisposition, the importance of injury pattern, and the impact of potentially modifiable postinjury care [1]. TXA inhibits plasminogen (the proenzyme) binding to plasmin (ie, blocking activation of plasmin), and it also inhibits the binding of plasmin (the active form) to fibrin (ie, blocking fibrinolysis). Hence, the mechanism of action is to stabilize existing clots rather than promoting new clot formation [51]. This is an important distinction, as it emphasizes that TXA is an antifibrinolytic and not an antihemorrhagic agent [29]. In hemorrhagic trauma patients three different phenotypes of fibrinolytic responses of the body to massive trauma have been described: hyperfibrinolysis, physiologic fibrinolysis, and hypofibrinolysis (also referred to as “fibrinolytic shutdown”) [28]. An increased risk for fibrinolytic shutdown among severely injured trauma patients receiving TXA has been described (RR = 1.65 [95% CI, 11.10–1.64]; *p* = 004) [52]. Thus, the nondiscriminative and liberal administration of an antifibrinolytic agent to a patient population that could be largely in a hypofibrinolytic state does not seem to be physiologically or pharmacologically sound and would potentially expose patients to a higher risk for thromboembolic complications [29].

Reports on VTE risk after TXA administration in trauma patients have been inconsistent and many studies do not show a significantly increased incidence of VTE after TXA [9, 16, 17, 19, 24, 53]. However, together with other publications we report an independently associated increase in VTE risk with the administration of TXA in trauma patients [1, 20, 22, 27, 31, 40, 47]. Unique to our finding is the independently related increase with every single dose given at different treatment periods, including prehospital and emergency department intervals. Additionally, our patient population, receiving at least two administrations of TXA, comprises more than 1,000 trauma patients and exceeds the number of patients reported on so far with dose dependent VTE risk of TXA [31, 38, 40]. As far as the amount of TXA in a single-dose and/or repetitive administrations is concerned, studies and regimens vary substantially [31, 35, 38, 46, 54]. This is, why results of different studies cannot be readily discussed or applied interchangeably.

Gunn et al. [38] report on different dosing strategies of TXA in trauma patients and see increasing VTE rates in patients receiving TXA. This study comprised 525 trauma patients treated in one Level-1 trauma center over an 11-year period. All patients received TXA intravenously, either 1 g or 2 g (double-bolus) or an initial 1 g bolus and consecutive 1 g infusion over 8 h i.v. They state VTE rates of bolus + infusion or double-bolus regimen in comparison to single-dose administration of TXA to be 8%, 7% and 4%, respectively. Within their reported patient sample no statistical difference was found between TXA groups with *p* = 0.31. Yet, their reported percentages are similar to ours with TXA single dose VTE rate 4.8% and 5.2% (prehospital; ED), as well as double-dose 8.5% (prehospital + ED), respectively. In our larger sample we were able to detect a statistically related significant VTE risk inherent to every dose of administered TXA.

The STAAMP study [46] assessed the administration of TXA in trauma patients during prehospital and early hospital treatment. 927 patients were originally enrolled in the RCT prehospitally and data from 903 patients continuing the hospital treatment were finally analyzed. The TXA treatment was either none, or 1 g i.v. at any of 3 consecutive treatment phases (prehospital bolus, hospital bolus, hospital infusion over 8 h). This generated treatment groups of no-TXA and either 1 g-, 2 g-, or 3 g-TXA doses. There was no statistically significant reduction in 30-day mortality found for TXA-treatment versus no-TXA. Also, the prehospital administration of TXA did not result in a higher incidence of thrombotic complications. Several facts of this study have to be taken into consideration when compared to our results. In the statistical workup, all patients treated with at least one dose of TXA were assigned to the treatment arm. No differentiation was made according to multiple administration of TXA. Patients enrolled in the STAAMP trial had a median ISS of 11 (IQR 4–22) in the placebo and 13 (IQR 5–22) in the treatment arm. In our study 67.4% of our patients had an ISS ≥ 16. In the STAAMP trial prehospital median SBP was 126 (IQR 87–148) mmHg in the placebo and 123 (IQR 88–143) mmHg in the treatment arm. Transfusion of PRBC was rarely necessary in both arms. In contrast, we report 7.3% of our patients had been in shock (SBP ≤ 90 mmHg) prehospitally and a transfusion rate of 9.8% in all patients (1.1% mass transfusions). Overall, patients enrolled in the STAAMP trial seemed to be less severely injured, compared to our patients. Interestingly, when comparing tranexamic acid effect stratified by time to treatment and qualifying shock severity in a post hoc comparison of the STAAMP trial, 30-day mortality was significantly lower when tranexamic acid was administered within 1 h of injury and patients with severe shock (SBP ≤ 70 mmHg) who received tranexamic acid demonstrated a significant lower 30-day mortality compared with placebo [46]. As far as the effect of TXA on VTE incidence is concerned, no post-hoc comparison was undertaken. Yet, overall 25 VTE (5.6%; 13 PE, 12 DVT) in the TXA and 14 VTE (3.1%; 7 PE, 7 DVT) in the placebo arm are documented, albeit without statistically significant difference. Comparing these results to ours, we report an overall VTE incidence of 5.5% in the TXA treated and 2.3% in the no-TXA groups, yet we were able to additionally differentiate the associated significant contribution of any additional TXA administration to VTE risk in our study of severely injured patients. Interestingly, Cole et al. published data where 385 severely injured patients (ISS > 15) were treated at a hospital and 160 patients received TXA as part of major hemorrhage control. No statistical difference for VTE incidence was found for TXA treatment when the entire cohort or non-shocked (base deficit ≤ 6 mEq/L) patients were analyzed. However, in the subgroup of shocked patients (base deficit ≥ 6 mEq/L measured at admission to hospital) there was a fourfold increase in thromboembolic events in the TXA group (no-TXA: 2% vs. TXA: 8%, *p* < 0.01) [11].

In several studies, risk factors for VTE in multiply injured trauma patients have been established. These include e.g. older age, obesity, male sex, higher ISS, pelvic injury, lower extremity injury, spinal injury, delayed VTE prophylaxis and need for surgery [1]. In our collective, the analyzed groups of patients suffering from VTE and without VTE significantly differ in almost all of the above known factors. This is an imminent bias of retrospective data acquisition, when established protocols favor patient treatment. TXA has been recommended in severely injured hemorrhagic patients or patients at risk of significant bleeding [12, 13, 30]. It may be argued that our patient cohort treated with TXA is prone to VTE just because of the underlying magnitude of the injury and subsequent treatment (including different resuscitation strategies), yet this has to be debated in all studies retrospectively evaluating TXA and VTE complication [29]. To address this issue, we did not only evaluate the incidence rate of VTE with increasing administrations of TXA, but also underwent multivariate logistic regression adjusted for the known influencing covariates—and found VTE to be independently related to applications of TXA.

In an attempt to possibly explain our finding, as mentioned above, several authors have described a phenomenon called ‘fibrinolytic shutdown’ in traumatized patients [55, 56] where administration of an antifibrinolytic agent would potentially expose patients to a higher risk for thromboembolic complications [29]. The routine use of viscoelastic techniques have allowed to describe specific fibrinolytic phenotypes (hyperfibrinolysis versus shutdown) and to establish a relationship of these phenotypes with outcome. Several authors have tried to associate the presence of fibrinolytic shutdown and the incidence of thrombotic complications and mortality when TXA was given, some with positive results, some with negative results. The consequence is that some authors are now advocating for a more targeted approach when it comes to the administration of TXA in traumatized patients [52, 57]. The challenge being that viscoelastic techniques are known to have a very poor sensitivity to fibrinolytic activation and were not designed to assess different degrees of fibrinolytic activation [30].

### Limitations

The current study has several limitations. The number of patients who received three consecutive TXA administrations entering the analysis is small. Thus, the reported confidence interval is rather large. However, with a comparable OR 1.50 (likewise the OR of one and two administrations) it can be assumed there are correlated higher VTE incidences with three times dosing of TXA.

The administered TXA dose, injection time (bolus or infusion) and exact time of application were not recorded in the TraumaRegister DGU® (TR-DGU). Therefore, we comment on the cumulative timepoints when patients received TXA.

Clinically inapparent VTE and VTE diagnosed after hospital discharge were not part of the documentation of the TR-DGU. Therefore, documented incidences represent only clinically relevant VTE complications. Supposedly, the incidence of thromboembolic events in our study population might be underestimated. Furthermore, no information can be given regarding different mechanism or substances of thromboprophylaxis, as well as the time when thromboprophylaxis was started, since this information was not entered in the TR-DGU.

Since the TR-DGU only encompasses data from the prehospital and inpatient interval of treatment, data after discharge is not sought after and not entered. Therefore, we cannot comment on events ensuing after hospital discharge (e.g. VTE, death, etc.).

## Conclusions

In our study we report on an associated imminent risk of thromboembolic events in multiply injured patients with every single time tranexamic acid is administered along the treatment intervals “prehospital”, “emergency department” and “operating room” until the initial phase of treatment on the intensive care unit”.

However, TXA should not be withheld from patients with severe traumatic injury and hemorrhage, but thromboembolism screening should be considered for patients receiving at least one dose of TXA intravenously. Before a repetitive TXA dose is administered checking for indication is advised and in-hospital thrombelastography to define the coagulopathic pattern and screen for supposedly benefitting patients seems reasonable. Especially in multiply injured patients receiving repeated administrations of TXA starting a thromboprophylaxis, as soon as possible after the traumatic bleeding disorder is controlled, is important.

## Data Availability

The datasets used and/or analyzed during the current study are available from the corresponding author on reasonable request and with permission of the TraumaRegister DGU® (TR-DGU) of the German Trauma Society (DGU).

## Abbreviations

AIS: Abbreviated Injury Score
CI: Confidence interval
DGU: Deutsche Gesellschaft für Unfallchirurgie
DVT: Deep vein thrombosis
ED: Emergency department
GCS: Glasgow Coma Scale
ICU: Intensive care unit
IQR: Interquartile range
ISS: Injury Severity Score
MI: Myocardial infarction
OR: Odds Ratios
PE: Pulmonary embolism
PRBC: Packed red blood cells
SBP: Systolic blood pressure
SD: Standard deviation
TR-DGU: TraumaRegister DGU®
TXA: Tranexamic acid
VTE: Venous thromboembolism
